# SKI2 mediates degradation of RISC 5′-cleavage fragments and prevents secondary siRNA production from miRNA targets in *Arabidopsis*

**DOI:** 10.1093/nar/gkv1014

**Published:** 2015-10-12

**Authors:** Anja Branscheid, Antonin Marchais, Gregory Schott, Heike Lange, Dominique Gagliardi, Stig Uggerhøj Andersen, Olivier Voinnet, Peter Brodersen

**Affiliations:** 1 Department of Biology, University of Copenhagen, Ole Maaløes Vej 5, DK-2200 Copenhagen N, Denmark; 2 Swiss Federal Institute of Technology (ETH) Zürich, Department of Biology, LFW D17/D18, Universitätsstrasse 2, CH-8092 Zürich, Switzerland; 3 Institut de Biologie Moléculaire des Plantes du CNRS, 12 Rue du Général Zimmer, F-67084 Strasbourg Cedex, France; 4 Department of Molecular Biology, University of Aarhus, Gustav Wieds Vej 10, DK-8000 Aarhus C, Denmark

## Abstract

Small regulatory RNAs are fundamental in eukaryotic and prokaryotic gene regulation. In plants, an important element of post-transcriptional control is effected by 20–24 nt microRNAs (miRNAs) and short interfering RNAs (siRNAs) bound to the ARGONAUTE1 (AGO1) protein in an RNA induced silencing complex (RISC). AGO1 may cleave target mRNAs with small RNA complementarity, but the fate of the resulting cleavage fragments remains incompletely understood. Here, we show that *SKI2*, *SKI3* and *SKI8*, subunits of a cytoplasmic cofactor of the RNA exosome, are required for degradation of RISC 5′, but not 3′-cleavage fragments in *Arabidopsis*. In the absence of SKI2 activity, many miRNA targets produce siRNAs via the RNA-dependent RNA polymerase 6 (RDR6) pathway. These siRNAs are low-abundant, and map close to the cleavage site. In most cases, siRNAs were produced 5′ to the cleavage site, but several examples of 3′-spreading were also identified. These observations suggest that siRNAs do not simply derive from RDR6 action on stable 5′-cleavage fragments and hence that SKI2 has a direct role in limiting secondary siRNA production in addition to its function in mediating degradation of 5′-cleavage fragments.

## INTRODUCTION

Small non-coding RNAs constitute an important element of post-transcriptional gene control in many biological systems. In eukaryotes, different classes of 20–24 nt RNAs are derived from double stranded RNA (dsRNA) via cleavage by Dicer ribonucleases. Small RNAs join proteins of the ARGONAUTE (AGO) family to form RNA induced silencing complexes (RISCs). RISCs use base pairing to target complementary mRNA for repression, in plants either via AGO-catalyzed transcript cleavage (‘slicing’) or via translational repression (1–5). Two main classes of small RNA are implicated in post-transcriptional gene control in plants. microRNAs (miRNAs) are generated as single species from short, imperfectly paired hairpin transcripts, while short interfering RNAs (siRNAs) accumulate as populations of small RNA species from longer, perfectly double stranded RNA molecules (6). Both types of small RNA may use the same effector, AGO1, for target regulation, but their biogeneses follow distinct pathways (6–9).

siRNA production may also result from amplification loops in which a target transcript of a primary small RNA becomes a substrate for RNA dependent RNA polymerase (RDR) resulting in production of dsRNA that is in turn diced into new waves of siRNAs, termed secondary siRNAs (10). Several RDRs are encoded in plant genomes. RDR1 and RDR6 have been implicated in post-transcriptional silencing pathways, while RDR2 plays a key role in transcriptional gene silencing (11–16). RDR-dependent siRNA amplification, or transitivity, is crucial in antiviral defence (13,17), and while transgenes are also prone to RDR6-mediated transitivity, most endogenous transcripts are largely refractory to transitivity (10,18). In particular, the majority of protein-coding miRNA targets do not sustain transitivity, although a specific subset, predominantly targeted by multiple small RNAs, produce abundant RDR6-dependent transitive siRNAs (19). The precise basis for the widespread suppression of transitivity on endogenous transcripts is unclear, in particular because increased abundance of uncapped and non-polyadenylated transcripts, such as those generated by miRNA-directed RISC cleavage, correlates with onset of RDR6-dependent transitivity (20,21). Part of the reason may be that RISC cleavage fragments are subject to rapid degradation, thus highlighting direct competition between decay pathways and RDR6 as a mechanistic key to restriction of transitivity. Rigorous tests of this idea clearly require identification of the decay pathways involved in degradation of RISC cleavage fragments.

RISC 3′-cleavage fragments can be detected in wild type backgrounds, but their abundance is increased in mutants of the 5′-3′-exoribonuclease XRN4 (22). *xrn4* mutants are exceptionally prone to RDR6-mediated transgene silencing (20), and generate siRNAs from many endogenous loci that do not produce siRNAs in wild type (23). In this manuscript, we term such siRNAs ‘illegitimate siRNAs’, because of the existence of mechanisms that prevent their production in wild type.

RISC 5′-cleavage fragments turn over rapidly judging from their poor detection compared to 3′-cleavage fragments (1), but degradation pathways responsible for their decay remain ill-defined in higher plants. 5′-cleavage fragments are uridylated at the 3′ ends by the AGO1-associated uridyltransferase HESO1, and uridylation may stimulate their degradation, since a few 5′-cleavage fragments display modest overaccumulation in *heso1* mutants (24). XRN4 may contribute to degradation of 5′ cleavage fragments, but no components involved in 3′-5′ exonucleolytic pathways have been isolated thus far (24). In particular, a viable mutant in the CSL4 subunit of the RNA exosome, as well as mutants in homologues of the exosome-associated 3′-5′ nuclease Rrp6, do not show higher levels of a RISC 5′ cleavage fragment (24). In the green alga *Chlamydomonas reinhardtii*, degradation of 5′-cleavage fragments produced by siRNA-containing RISC also depends on an adenyl/uridyl transferase (MUT-68), but in this system, the RNA exosome and Rrp6 are also required for degradation of 5′-cleavage fragments (25). Similarly, in *Drosophila* cell cultures, 5′-cleavage fragments of reporter mRNA cleaved by Dm-Ago2 programmed with transfected siRNA are degraded by a pathway that requires the RNA exosome as well as the DExH-box helicase SKI2, the tetratricopeptide-repeat protein *SKI3*, and *SKI8* (26). The exosome and associated nucleases degrade a wide variety of RNA substrates by 3′-5′exonucleolysis, and requires a series of cofactors for different classes of substrates (27). *SKI2*, *SKI3* and *SKI8* form a heterotetrameric complex in yeast (28,29), and structural studies suggest that they form a regulatory particle feeding RNA substrates to the exosome similar to the lid of the proteasome in protein degradation (30). The SKI2–3–8 complex is cytoplasmic in yeast and *Arabidopsis* (28,31), and complexes with DExH helicases homologous to SKI2 (MTR4 and, in plants, also HEN2) play roles as exosome cofactors in the nucleus (32–34). It is unclear, however, to what extent results on the involvement of exosome cofactors in RISC 5′-cleavage fragment degradation obtained in the *Drosophila* Ago2-dependent system are applicable to plants, not only because of the evolutionary distance between plants and insects, but in particular because *Drosophila* Ago2, in contrast to *Drosophila* Ago1, is phylogenetically very different from all plant AGOs.

In this study, we show that *SKI2*, *SKI3* and *SKI8* are required for degradation of 5′-fragments generated by miRNA-guided RISC cleavage in *Arabidopsis*. In the absence of this degradation pathway, a miR171-targeted GFP reporter generates high amounts of RDR6-dependent siRNAs exclusively 5′ to the cleavage site. Several endogenous miRNA targets also produce low-abundant illegitimate transitive siRNAs 5′ to the miRNA target site in a *ski2* mutant, indicating that SKI2 protects against miRNA-triggered transitivity. Remarkably, examples of exclusive 3′-spreading of transitive siRNA in *ski2* mutants were also identified, suggesting that the trigger of transitivity is not simply accumulation of 5′-cleavage fragments.

## MATERIALS AND METHODS

### Plant material and growth conditions

The GFP171.1 line is in accession C24 and carries the *sde1–1* mutation in RDR6 (12). *mad7/ski2–4* was isolated from an ethylmethane sulfonate mutagenized population of GFP171.1 as previously described (4). All analyses reported in this manuscript were performed on lines backcrossed twice to GFP171.1. T-DNA insertion lines in *SKI2* (AT3G49690; *ski2–2* (Salk_129982), *ski2–5* (Salk_118579)), *SKI3* (AT1G76630; *ski3–5* (GK_140B07_012876)) (35) and *SKI8* (AT4G29830; *ski8–1* (Salk_083364)) were ordered from the Nottingham Arabidopsis Stock Centre. For all seedling analyses, plants were grown for 16 days on 1xMurashige and Skoog medium in petri dishes under a 16 h light/8 h darkness regime. For analysis of inflorescences, seedlings were germinated on 1xMS plates, transferred to soil and grown in a Percival growth chamber at 70% relative humidity under a 16 h light (21C)/8 h darkness (16C) regime.

### Mutant genotyping

T-DNA insertion lines were genotyped with two chromosomal primers flanking the insertion site for presence of wild type allele, and an outward left border primer and a flanking chromosomal primer for the T-DNA allele. CAPS markers were used for genotyping *ski2–4* (Sau3A) and *sde1-1* (StyI). Primer sequences are listed in Supplementary Table S4.

### Construction of double mutants

To obtain double homozygous combinations of *SKI2* and *RDR6* mutant and wild type alleles, *ski2–4/rdr6* was crossed to C24 wild type. Kanamycin selection was applied in F2 to select for the GFP171 transgene, and all mutant combinations were identified by polymerase chain reaction (PCR) in F2 using genotyping assays described above. Mutant genotypes were confirmed by PCR in F3. *ski2–4*/*rdr6*/GFPnomiR was generated by crossing *ski2–4/rdr6*/GFP171.1 to *SKI2/rdr6*/GFPnomiR (4). The F2 generation was PCR-screened for loss of GFP171.1 as described in (4), and *ski2–4* and *rdr6* point mutations were detected by PCR and restriction digest as described above.

### Identification of the MAD7/SKI2 gene

*ski2–4/rdr6* in GFP171.1 was crossed to *rdr6–3* (accession Ler). GFP northern blots of 12 individuals of two F2 families showed that the segregation of the presence of the 5′ cleavage fragment of GFP mRNA was consistent with a single recessive mutation in the mixed ecotype background. 414 kanamycin resistant seedlings were transferred to soil, and RNA was extracted from inflorescences of individual F2 plants and analyzed by northern blot with a GFP probe synthesized by random priming from a template amplified with the primers GFP1 and GFP4 from genomic DNA extracted from GFP171 (see Supplementary Table S4 for primer sequences). Genomic DNA from each of the 99 individuals selected to be mutant by northern blotting was prepared, and an equal amount of DNA from each sample was mixed. The pool of DNA was sequenced as 2 × 101 bp paired-end reads on an Illumina platform. Illumina reads were mapped to the *Arabidopsis* Col-0 reference genome using bowtie version 0.12.3 (36). SHOREmap version 1.1 was then used to identify the candidate interval and causal mutation as previously described (37). The markers used for identification of the candidate interval were a set of C24/Col-0 SNPs from the 1001 genomes project (http://1001genomes.org/data/MPI/MPISchneeberger2011/releases/2010_08_17/strains/C24/) (38), and re-sequencing data from an additional mutant in the GFP171.1 background was used to further reduce the number of false positives resulting from unannotated C24/Col-0 polymorphisms.

### Western blotting

For preparation of total protein extracts, 100 mg of finely ground tissue was mixed with 500 μl NuPAGE LDS sample buffer [106 mM Tris HCl 141 mM Tris Base 2% LDS 10% Glycerol 0.51 mM EDTA 0.22 mM SERVA Blue G250 0.175 mM Phenol Red pH 8.5, 5 mM fresh DTT], heated for 10 min at 70ºC and centrifuged to pellet plant cell debris. The supernatant was transferred into a new tube and 10 to 20 μl of the lysate were subjected to SDS-PAGE. GFP antibodies, a kind gift from David Gilmer, have been described (4). Detection of horseradish peroxidase (HRP)-conjugated secondary antibodies (Sigma) was performed by enhanced chemiluminescence (ECL) reaction (Roche Lumilight).

### RNA extraction

Total RNA from plant tissue was extracted with TRI reagent (Invitrogen) according to the manufacturer's instructions. The RNA was dissolved in 50% formamide for northern analysis.

### Analysis of mRNA cleavage fragments and mRNA levels

For cleavage fragment analysis, 20 μg of total RNA was loaded onto a 1% denaturing agarose gel and separated for 3–5 h at 120V. The RNA was blotted to an Amersham Hybond-NX nylon membrane (GE Healthcare Life Sciences) and UV-crosslinked (254 nm). After pre-hybridization in PerfectHyb^TM^ Plus Hybridization buffer (Sigma) for 1 h at 65ºC, the radioactively labeled probe (Prime-a-gene labeling kit, Promega) was hybridized overnight at 65ºC. Sequences for primers used for amplification of probe templates for GFP and endogenous miRNA targets are listed in Supplementary Table S4. After hybridization, the membrane was washed 3 times in 2xSSC (0.3 M NaCl, 30 mM sodium citrate), 0.1% SDS at 65ºC and developed by phosphorimaging. For Terminator (Epicentre) reactions, 10 μg of total RNA was incubated with 1 U Terminator (Epicentre) for 1 h at 30ºC. The reaction was terminated by phenol extraction and subsequent ethanol precipitation. The RNA was resuspended in 50% formamide and loaded with untreated RNA as control on a 1% denaturating agarose gel. Poly(A) RNA was purified from 10 μg of total RNA using Dynabeads Oligo(dT)_25_ (Life Technologies) according to the manufacturer's instructions.

### Analysis of small RNAs

10 μg RNA from total extracts was denatured at 95ºC for 10 minutes in sRNA loading buffer [20 mM HEPES pH7.8, 1 mM EDTA, 50% formamide, 3% glycerol and 0.01% bromophenol blue (BPB)], and separated in a denaturing 18% polyacrylamide gel. The RNA was blotted on an Amersham Hybond-NX nylon membrane (GE Healthcare Life Sciences) and crosslinked by 1-ethyl-3-(3-dimethylaminopropyl) carbodiimide (EDC) for 2 h at 60ºC (39). Radioactively end-labeled probes were hybridized to the membrane in PerfectHyb^TM^ Plus Hybridization buffer (Sigma) at 42ºC overnight. Membranes were washed 3x with 2xSSC, 2% SDS at 42ºC for 15 minutes, and developed by phosphorimaging.

### 3′ RACE analysis

3′-ends of GFP171 cleavage fragments were identified as described in (40). Briefly, 5 μg of total RNA was purified with Nucleospin RNA plant columns (Macherey Nagel), dephosphorylated with calf intestinal phosphatase (CIP, NEB) followed by ligation to the RNA adapter (5′P-CUAG AUGAGACCGUCGACAUGAAUUC-3′NH2) with T4 RNA ligase (Fermentas). After removal of excess primer with Nucleospin RNA plant columns (Macherey Nagel), the adapter-ligated RNA was used as a template for the reverse transcription reaction by SuperScript III reverse transcriptase (Invitrogen) with primer ADa (GAATTCATGTCGACGGTCTCA). Two nested PCR amplification of 30 cycles were performed using adapter primer ADa and GFP13 for the first PCR and primer ADb (CATGTCGACGGTCTCATCTAG) and GFP11 for the second PCR (Supplementary Table S4). PCR products were cloned into pGEM-Teasy and individual clones were Sanger sequenced.

### Construction of libraries for small RNA sequencing

For preparation of small RNA cDNA libraries for Illumina sequencing, 2 μg of total inflorescence RNA was used. All libraries were generated using the NEBNext Small RNA Library Prep Set (Multiplex) (New England Biolabs) according to manufacturer′s instruction with the following minor changes. After final PCR amplification, 30 nmol of DNA from each library with distinct barcodes was pooled and purified in a 3% metaphor agarose (LONZA) gel stained with ethidium bromide. A gel piece in the expected size range was cut out and DNA was purified using MinElute Gel extraction kit (Qiagen). The purified DNA was analyzed using an Agilent Bioanalyzer and sequenced on an Illumina platform.

### Analysis of Illumina small RNA sequence reads

The ncPRO pipeline (41) was used to process the sRNA-sequencing raw data, to map the reads against the *Arabidopsis thaliana* genome (version TAIR9 from www.arabidopsis.org) and to analyze the quality of deep sequencing globally. Unmapped reads were aligned against the GFP171.1 sequence using the Bowtie program (36).

A scanning windows strategy was used to address the differential accumulation of sRNAs along the full genome of *Arabidopsis thaliana*. We counted the read numbers independently for each read size from 19 to 26 nt in windows of 300 nt overlapping by 150 nt along the entire genome. The differential expression for each window and each read size was analyzed with the R package edgeR (42). Statistics and figures representing small RNA sequencing data were computed with R.

### Fluorescence microscopy

Pieces of stem tissue from equivalent positions in the shoots of the different genotypes were embedded in 1% low-melt agarose, and thin sections were cut with a scalpel. GFP fluorescence was examined and photographed with a Leica MZ16F fluorescence stereomicroscope.

## RESULTS

### Isolation of the *mad7/ski2–4* mutant

**Figure 1.**
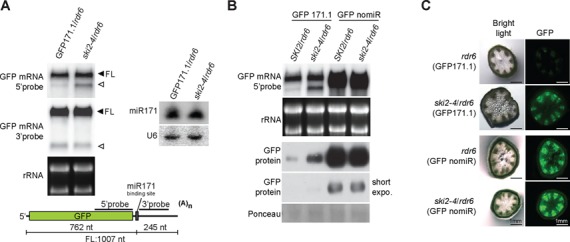
Isolation of the *ski2–4* mutant. (**A**) Left panel, northern blot analysis of 20 μg of total inflorescence RNA from GFP171.1/*rdr6* and *ski2–4/rdr6*. The same blot was hybridized consecutively to radiolabeled 5′ and 3′ probes as indicated in the schematic of the GFP171.1 transgene. FL, full length. rRNA, ethidium bromide stained ribosomal RNA in gel prior to blotting. Right panel, northern blot of total inflorescence RNA probed with end-labeled oligonucleotides complementary to miR171 and U6. (**B**) RNA and protein analysis from inflorescences performed as described in (A) above. (**C**) Fluorescence microscopy images of GFP accumulation in cross sections of stems of the indicated genotypes.

We previously described a forward genetic screen to select mutants with defects in miRNA biogenesis or action (4). Briefly, a *GFP* transgene harboring a miR171 target site immediately downstream of the stop codon (*GFP171.1*) was constitutively expressed in an *rdr6* mutant background to avoid miR171-triggered transitivity (Figure 1A, (43)). GFP171.1 is silenced via mRNA degradation and translational repression by endogenous miR171 in this system, and we previously reported on isolation of mutants with reactivated GFP, defining miRNA biogenesis deficient (*dcl1–12* and *hen1–7*) and miRNA action deficient (*mad1-mad6*) classes (4). *mad7* (renamed *ski2–4* as described below, and referred to as *ski2–4/rdr6* to specify the *rdr6* mutant background) was selected upon continued screening based on GFP reactivation in seedlings, and caught our interest, because northern blots showed the appearance of a shorter GFP RNA in addition to the full-length transcript in this mutant (Figure 1A, left panel). This pattern was not observed in previously isolated mutants. miR171 levels were not affected in *ski2–4/rdr6* (Figure 1A, right panel). Although the mutant was isolated on the basis of its GFP accumulation in seedlings, we did not detect elevated GFP protein levels in *ski2–4/rdr6* seedlings after two backcrosses to the GFP171.1 parental line (Supplementary Figure S1). On the other hand, inflorescences of *ski2–4/rdr6* maintained elevated GFP levels compared to the parental GFP171.1 line, and since an otherwise identical, but non-targeted GFP line (GFP nomiR) did not overaccumulate GFP protein in *ski2–4/rdr6* inflorescences compared to *SKI2/rdr6* segregants, we conclude that a mutation in the *ski2–4/rdr6* line affects repression of GFP171.1 expression by miR171 (Figure 1B). The GFP upregulation in *ski2–4/rdr6* specifically in GFP171.1, but not in GFP nomiR backgrounds, was also visible by fluorescence microscopy of cross sections of stem tissue (Figure 1C). We do not characterize the upregulation of GFP171.1 in *ski2–4*/*rdr6* further in this study, and focus here on understanding the appearance of the second species of GFP RNA revealed by northern blotting.

### RISC 5′ cleavage fragments accumulate in *ski2–4/rdr6* mutants

**Figure 2.**
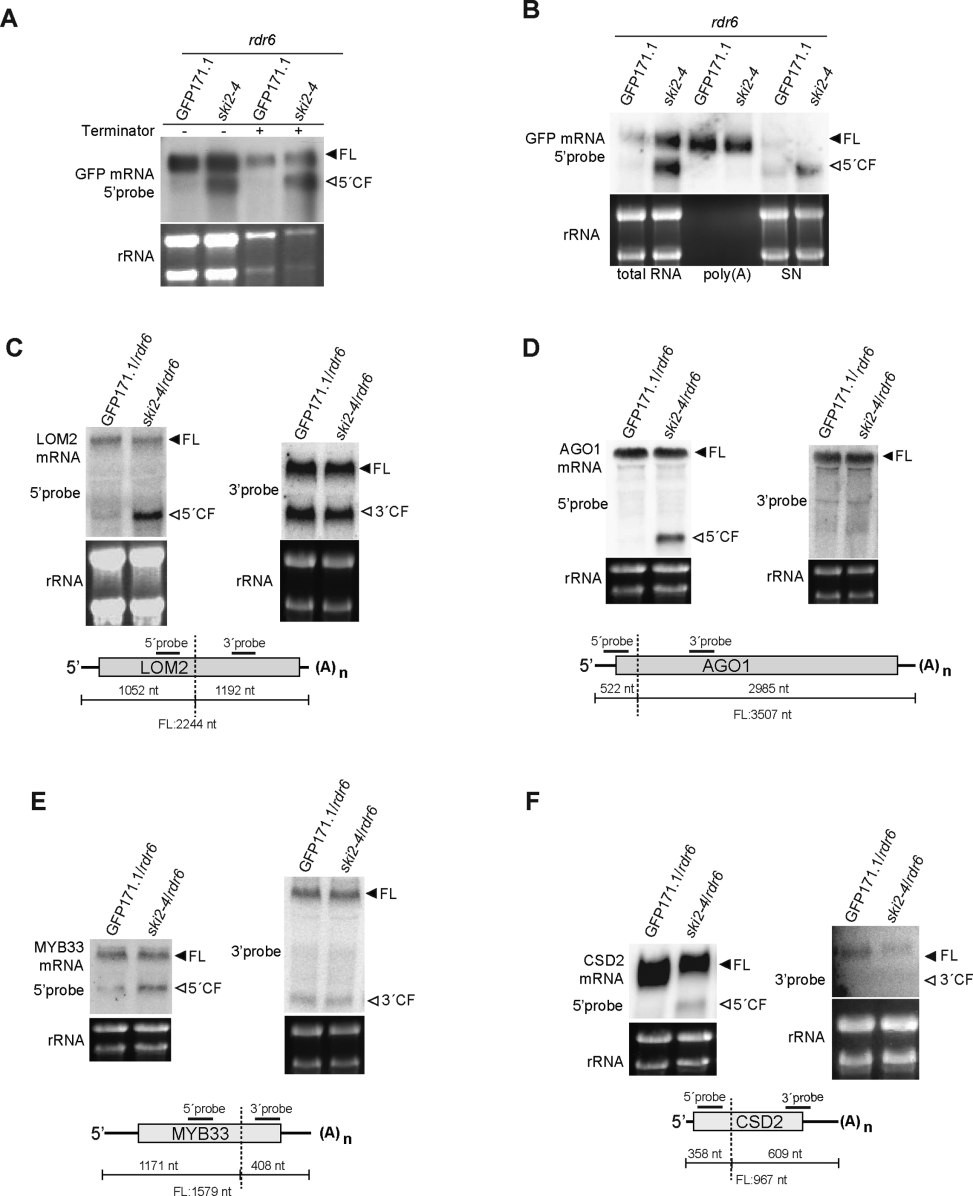
Characterization of 5′-cleavage fragments in *ski2–4*. (**A**) 10 μg of total RNA was incubated in Terminator buffer with or without enzyme, and the RNA was analyzed by northern blot hybridization to a radiolabeled 5′ GFP probe (Figure 1A). (**B**) Fractionation of poly(A)+ RNA using oligo(dT) coupled beads. 10 μg of total RNA was used as input. The unbound supernatant fraction (SN) was ethanol precipitated and analyzed in parallel with bound and total fractions by northern blot as in (A). (**C**–**F**) Northern blot analyses of 20 μg of inflorescence RNA. Radiolabeled probes specific to either 5′ or 3′ parts of LOM2, AGO1, MYB33 and CSD2 mRNAs with respect to miRNA cleavage sites were hybridized to six northern membranes prepared from the same batch of inflorescence RNA. 5′ and 3′ probes for each transcript were hybridized to different membranes. AGO1 and MYB33 5′-probes were hybridized consecutively to the same northern membrane, while independent membranes were prepared for hybridizations to LOM2 5′, LOM2 3′, CSD2 5′ and CSD2 3'-probes. AGO1 and MYB33 3′-probes were hybridized consecutively to the same membrane.

The size of the second GFP RNA species estimated from its migration rate relative to 18S and 28S rRNAs corresponded to the 5′-cleavage fragment generated by RISC. Three additional observations are consistent with this interpretation. First, the fragment was detectable with a GFP coding sequence probe 5′ to the cleavage site, but not with a 3′ UTR specific probe 3′ to the cleavage site (Figure 1A). Second, it was resistant to treatment with the Terminator 5′-3′ exoribonuclease, suggesting that it was capped (Figure 2A). Third, in contrast to full length GFP mRNA, it remained in solution upon incubation with oligo(dT)-coupled beads, indicating that it did not contain a poly(A) tail (Figure 2B). To test whether *ski2–4/rdr6* generally accumulated endogenous 5′-cleavage fragments generated by RISC cleavage, we analyzed four miRNA targets by northern blotting, LOM2 (miR171), AGO1 (miR168), MYB33 (miR159) and CSD2 (miR398; Figure 2C–F). In all cases, we detected increased accumulation in *ski2–4/rdr6* of mRNA fragments with sizes matching 5′ cleavage fragments judging by their migration rates relative to 18S and 28S rRNAs. Probes complementary to regions 3′ to miRNA cleavage sites did not detect any RNA species with increased accumulation in *ski2–4/rdr6* (Figure 2C–F). We conclude that 5′ cleavage fragments of miRNA targets accumulate in *ski2–4/rdr6* mutants, either because of enhanced biosynthesis or because of impaired degradation. Two observations argue that impaired degradation causes accumulation of 5′-cleavage fragments in *ski2–4/rdr6*. First, since the synthesis rate of 5′-cleavage fragments is proportional to the RISC cleavage constant, increased synthesis rate would be expected to also lead to increased accumulation of 3′-cleavage fragments. We did not observe different levels of 3′-cleavage fragments between GFP171.1/*rdr6* and *ski2–4*/*rdr6* (Figures 1A and 2C–F). Second, increased RISC cleavage of a miR171 target by transient miR171 overexpression in *N. benthamiana* did not lead to overaccumulation of 5′-cleavage fragments (1).

### Tailing of 5′ cleavage fragments is intact in *ski2–4/rdr6*

To test whether *ski2–4/rdr6* is affected in uridylation of 5′-cleavage fragments, we isolated and sequenced the 3′-ends of these fragments from *ski2–4/rdr6* and from the GFP171.1 parental line by a 3′-RACE approach. These analyses showed that a large fraction of 5′-cleavage fragments isolated from *ski2–4/rdr6* contained oligouridine or oligoadenosine tails with occasional occurrence of cytidines (Table 1). These data show that tailing of 5′-cleavage fragments in the form of uridylation and adenylation is intact in *ski2–4/rdr6*, but they do not allow conclusions to be drawn on whether tailed species are more prevalent in *ski2–4/rdr6* than in wild type.

**Table 1.** Summary of 3′-RACE analysis of the GFP171.1 5′-cleavage fragment

Number of 3′-RACE clones containing			
Genotype	5′ cleavage fragments	Tailed species	Tails identified
GFP171.1	17/50	2/17	U(1)
*SKI2/rdr6*			A(1)
			
			U(4), UU(1), UUA(7),
GFP171.1	81/81	26/81	U_4_(2), UCUAU_4_(2),
*ski2-4/rdr6*			U_5_CU(2), U_11_(1), U_12_(1)
			A(1), AA(3), AAC(1)
			C(1)

The different efficiency in cloning 5′-cleavage fragments between GFP171.1/*rdr6* and GFP171.1/*ski2–4/rdr6* is likely to be due to higher abundance in GFP171.1/*ski2–4/rdr6*. Most of the clones not matching the 5′-cleavage fragment in GFP171.1/*rdr6* contained cDNA corresponding to the uncleaved GFP mRNA.

### The *MAD7* gene encodes the DExH box helicase SKI2

**Figure 3.**
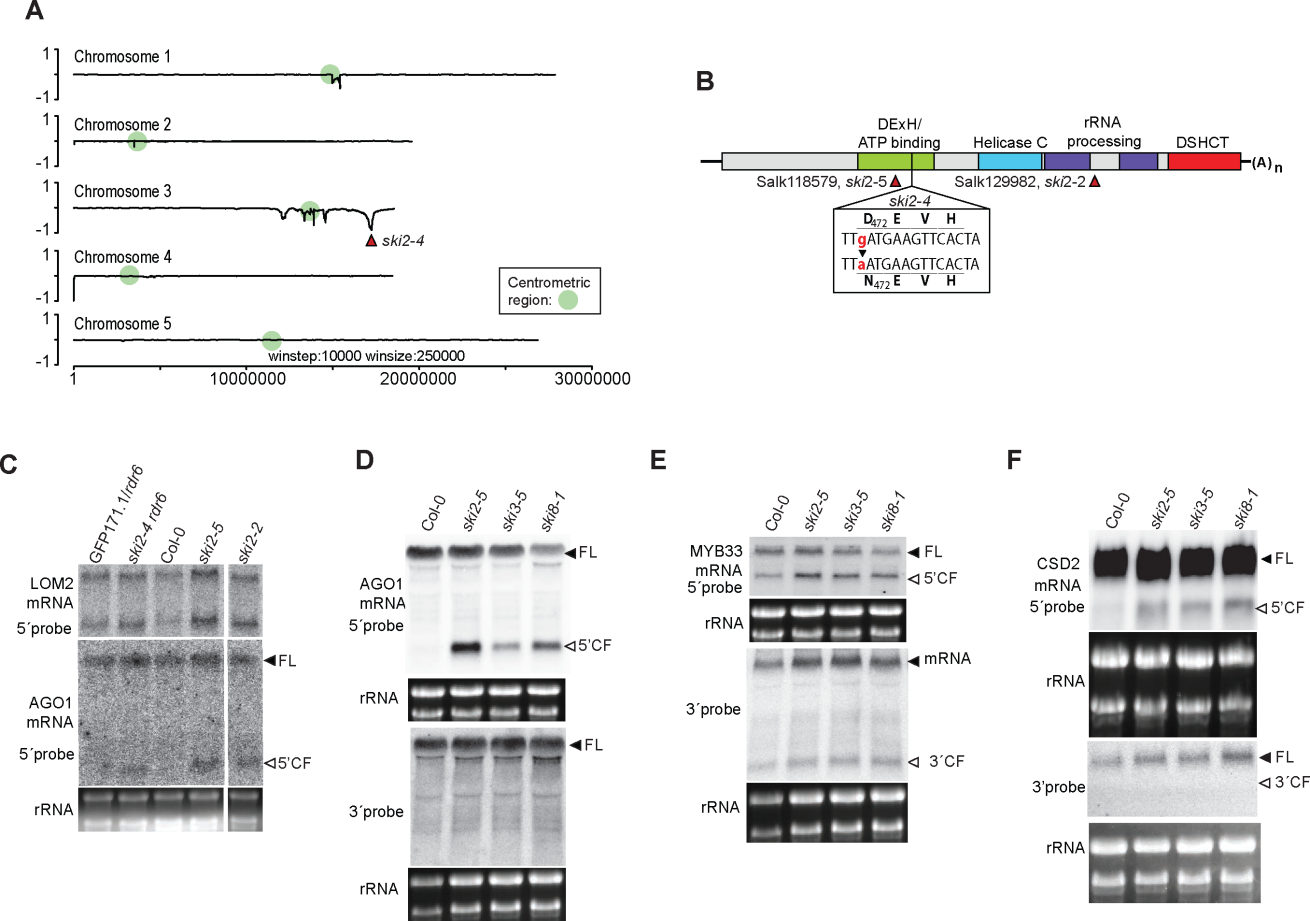
Identification of the *SKI2* gene. (**A**) SHOREmap allele-frequency plot of a pool of genomic DNA sequences obtained from 99 *ski2–4* mutants selected in the F2 population of a *ski2–4/rdr6* (C24) x *rdr6–3* (Ler) mapping population. Positive and negative values indicate enrichment of Ler and C24 alleles, respectively. (**B**) Schematic diagram of the SKI2 gene, indicating functional domains (Pfam nomenclature) and the positions of the *ski2–4* point mutation and the *ski2–2* and *ski2–5* T-DNA insertions. DExH and Helicase C, domains typical of RNA helicases; rRNA processing, domains necessary for ribosomal RNA processing in the SKI2 homologue MTR4; DCHCT, C-terminal domain occurring in DOB1/SKI2/helY-like DExH-box helicases. (**C**) Northern blot analysis of total inflorescence RNA hybridized with LOM2 and AGO1 probes as in Figure 2C, D. For LOM2, this set of samples exhibits unusually weak overaccumulation of the 5′-cleavage fragment in *ski2* mutant alleles compared to parental lines (the *ski2/*parental line ratio of 5′-cleavage fragment intensity normalized to full length transcript is 1.1 for the samples of the three *ski2* alleles shown here) (**D**–**F**) Northern blot analysis of total inflorescence RNA hybridized with AGO1, MYB33, and CSD2 5′ and 3′ probes as in Figure 2D–F. The same two membranes were used for the four AGO1 and MYB33 hybridizations (pairs of AGO1 5′/MYB33 5′ and AGO1 3′/MYB33 3′ hybridized to the same membranes), while two different membranes were used for CSD2 5′ and CSD2 3′ hybridizations.

We identified the *MAD7* gene by whole genome resequencing of pooled genomic DNA from 99 mutants selected by brute-force GFP northern blot screening of a mapping population for individuals with stable 5′-cleavage fragments. SHOREmap (37) analysis of sequence reads identified a clear, non-centromeric peak on chromosome 3 enriched for DNA of the parental accession C24 (Figure 3A). The orthologue of the yeast DExH box helicase *SUPERKILLER2* (*SKI2*, AT3G49690) was within 65000 bp of the peak, and a G-A transition was identified in this gene, altering the deeply conserved Asp residue in the DEVH box to Asn (D472N, Figure 3B, Supplementary Figure S2, Table S1). D472 coordinates the Mg^II^ ion in the Mg^II^-ATP complex bound by DEAD/DExH box helicases (44), and the D472N mutant encoded by *ski2–4* is therefore predicted to be catalytically inactive because of defective ATP binding. This mutation was the cause of stabilization of 5′-cleavage fragments in *ski2–4/rdr6*, because mutants with T-DNA insertions in the SKI2 gene in the Col-0 accession (*ski2–2* (SALK_129982) and *ski2–5* (SALK_118579)) also showed clear accumulation of 5′ cleavage fragments of several endogenous miRNA targets (Figure 3C, D–F).

### *SKI3* and *SKI8* are also required for degradation of 5′-cleavage fragments

We next tested the roles of the *Arabidopsis* homologues of *SKI3* (AT1G76630) and *SKI8* (AT4G29830) in degradation of RISC 5′ cleavage fragments. *SKI2*, *SKI3* and *SKI8* co-purify from *Arabidopsis* tissues suggesting that a SKI2–3–8 complex also exists in *Arabidopsis* (31,45). We isolated T-DNA insertion mutants in the *SKI3* and *SKI8* genes (Supplementary Figure S3), and analyzed accumulation of RISC 5′ cleavage fragments in these mutants in the RDR6 wild type background (accession Col-0). Similar to *ski2–4/rdr6*, the single mutants *ski2–5*, *ski3–5* and *ski8–1* showed clear overaccumulation of several endogenous 5′-cleavage fragments (Figure 3D–F), consistent with implication of the SKI2–3–8 complex in degradation of 5′-fragments generated by RISC cleavage.

### The 5′-cleavage fragment of GFP is a source of secondary siRNAs

**Figure 4.**
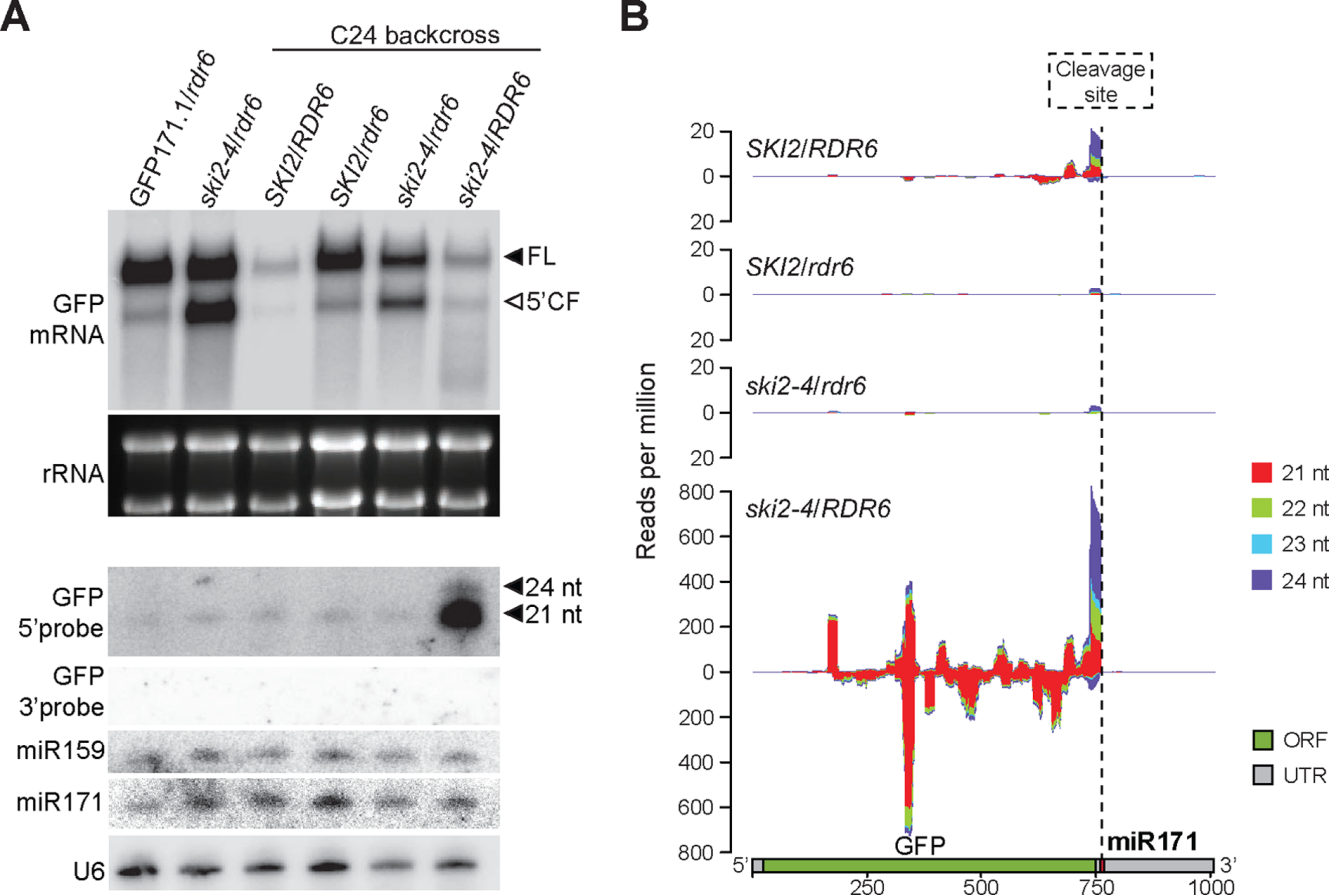
Transitive GFP siRNAs in *ski2–4*. (**A**) Top panel, inflorescence RNA analyzed by high molecular weight northern blot with a 5′ GFP probe as in Figure 1A. Bottom panel, same RNA analyzed by low molecular weight northern blot with consecutive hybridizations to 5′-GFP, 3′-UTR (as in Figure 1A), miR159 and miR171 probes in that order. miR159 and miR171 hybridizations demonstrate the integrity of the membrane after stripping of the 5′-GFP signal. The 3′-GFP probe had a comparable number of cpm/ml as the one used for the hybridization shown in Figure 1A. (**B**) Overview of siRNAs mapping to the GFP171.1 transcript in *SKI2/RDR6*, *SKI2/rdr6, ski2–4/rdr6* and *ski2–4/RDR6*. Ordinate, reads per million.

To test whether the 5′-cleavage fragment of GFP171.1 is a source of RDR6-dependent transitive siRNAs, we first outcrossed the *rdr6* mutation from *ski2–4*, and measured levels of GFP cleavage fragment and GFP siRNAs. The accumulation of the GFP cleavage fragment was less clear in the *ski2–4/RDR6* than in the *ski2–4/rdr6* background, suggesting that the fragment was processed by an RDR6-dependent pathway in the absence of SKI2 activity (Figure 4A, top panel). Consistent with this interpretation, highly abundant GFP siRNAs derived from the part 5′ to the miR171 cleavage site accumulated in *ski2–4*/*RDR6*, but not in *ski2–4/rdr6* or wild type (SKI2/RDR6) (Figure 4A, bottom panel). We note that no siRNAs were detected 3′ to the miR171 cleavage site (Figure 4A, bottom panel), indicating that inactivation of *SKI2* does not make the full-length GFP171.1 transcript a substrate for RDR6.

### Transitive siRNAs from miRNA targets in *ski2* mutants

**Figure 5.**
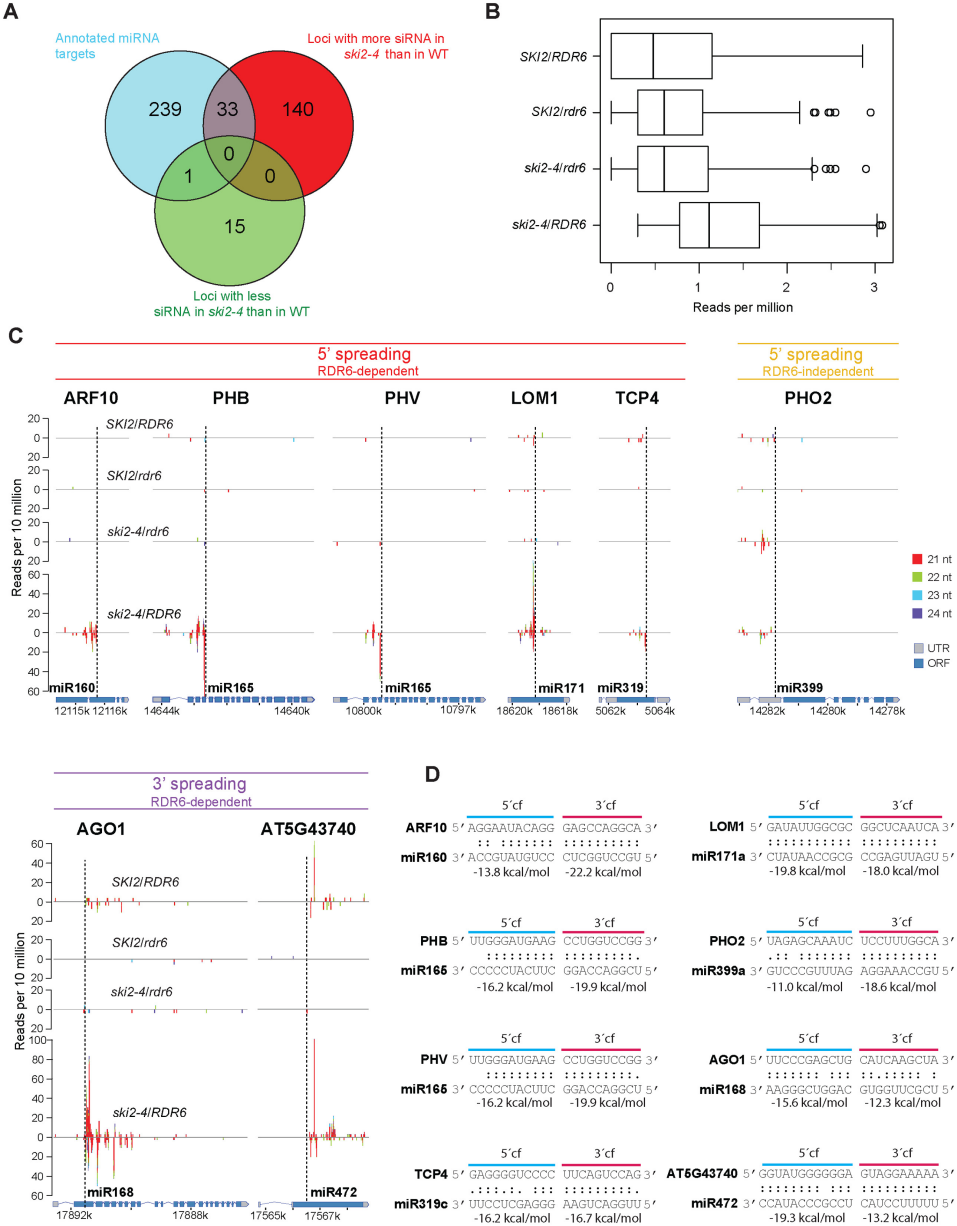
SKI2 limits transitivity on miRNA targets. (**A**) Venn diagram showing categories of loci with numbers of siRNA reads in *ski2–4/RDR6* significantly different from *SKI2/RDR6* (exact negative binomial test, *P* < 0.05). The enrichment of miRNA targets in loci with higher numbers of siRNA in *ski2–4/RDR6* is highly significant (Fisher-test: *P* < 2.2 × 10^−16^); this is not the case for the group of loci with lower numbers of siRNA in *ski2–4/RDR6* (Fisher-test: *P* = 0.09). (**B**) Boxplot of normalized mapped siRNA read counts in *SKI2/RDR6, SKI2/rdr6*, *ski2–4/rdr6* and *ski2–4/RDR6* for loci enriched in siRNAs in *ski2–4/RDR6* compared to *SKI2/RDR6*. Bars indicate medians, boxes indicate data between the first and third quartiles (Q1 and Q3, respectively) defining the interquartile range (IQR). Whiskers correspond to data between Q1 – 1.5 x IQR and Q3 + 1.5 IQR. Other values correspond to outliers and are represented as dots. (**C**) Examples of siRNA accumulation mapped to miRNA targets. Cleavage sites are indicated by dashed lines. PHO2 mRNA contains five closely spaced miR399 binding sites, all other dashed lines indicate the presence of a single miRNA binding site. The ordinate gives number of small RNA reads per 10 million, the abscissae depict TAIR9 coordinates corresponding to each gene. Small RNA libraries prepared from two replicates of each genotype were used for all analyses presented in this figure. (**D**) Strength of base pairing between miRNA and 5′ and 3′ cleavage fragments (cf). ΔGs were calculated for hybridization between free RNA strands with UNAFold (64) at 21°C, 150 mM NaCl, 5 mM MgCl_2_. PHO2 contains five closely spaced miR399 sites of which one is shown. Four of the five sites are similar to the one shown, the last site has stronger pairing to the 5′-cleavage fragment (See Supplementary Table S2). For LOM1, only pairing to the most abundant isomiR (miR171a) is shown. The other members of the miR171 family (miR171b/c, miR170) show stronger relative base pairing to the 3′-cleavage fragment (See Supplementary Table S2). For other targets, differences between isomiRs are negligible.

To investigate more broadly if SKI2 restricts production of transitive siRNAs, we sequenced small RNA libraries prepared from inflorescences of *SKI2/RDR6*, *ski2–4/RDR6*, *SKI2/rdr6* and *ski2–4/rdr6*. This analysis confirmed that GFP171.1 generates abundant RDR6-dependent 21-nt siRNAs exclusively on the 5′-side of the miR171 cleavage site upon inactivation of SKI2 (Figure 4B). A genome-wide search for loci producing significantly different levels of siRNAs in *ski2–4/RDR6* than in wild type identified a set of 189 loci (Figure 5A). In most cases (173/189), higher levels of siRNAs were detected in *ski2–4/RDR6* than in wild type (Figure 5A,B). Remarkably, although less than 1% of all *Arabidopsis* genes are known miRNA targets, they comprised 19% of the loci in this set, and were therefore strongly enriched (Fisher's test for statistical significance of enrichment of miRNA targets, *P* < 2.2 × 10^−16^, Figure 5A, Supplementary Table S2). It is possible that many of the remaining loci in this set produce transcripts not yet proven to be targets of miRNAs or endogenous siRNAs (Supplementary Table S3). In total, 14% of all known miRNA targets (including those that are not expressed in the tissue analyzed) produced significantly higher amounts of siRNAs in *ski2–4* mutants than in wild type (Figure 5A). This genome-wide analysis of aberrant siRNA production in *ski2–4/RDR6* suggests that SKI2 has a specific role in preventing transitivity initiated by miRNAs. Since transitivity often involves RDR6, we asked to what extent the production of siRNAs in *ski2–4/RDR6* from the 173 loci with more abundant siRNA in *ski2–4* mutants than wild type depended on RDR6. We treated all 173 loci as one group, and compared medians of siRNA read counts in this group between *ski2–4/RDR6* and *ski2–4/rdr6*. This analysis showed that the majority of siRNAs overaccumulating in *ski2–4* single mutants is RDR6-dependent (Figure 5B).

### Transitive siRNAs in *ski2–4* map close to cleavage sites on miRNA target transcripts

Mapping of transitive siRNAs onto individual miRNA target transcripts showed that they tended to accumulate on the 5′ side of miRNA target sites, that they were predominantly 21 nt in size, and that in all, but two cases, they were RDR6-dependent (Figure 5C, Supplementary Table S2). An example of this is provided by PHO2 where RDR6-independent siRNAs are located 5′ to the array of miR399-cleavage sites in the leader of the PHO2 mRNA (Figure 5C). Transitive siRNAs were low-abundant, and the peaks were restricted to a narrow region immediately adjacent to miRNA cleavage sites (Figure 5C). Remarkably, although most miRNA targets showed transitive siRNA accumulation exclusively 5′ to the miRNA cleavage site, five examples of siRNA accumulation exclusively 3′ to the cleavage site were also identified (Figure 5C, Supplementary Table S2). Two such examples, AGO1 (miR168) and the R gene AT5G43740 (miR472) are shown in Figure 5C. This pattern of siRNA accumulation was particularly noteworthy for AGO1, because the 5′ cleavage fragment was strongly stabilized while no stabilization of the 3′-cleavage fragment was detected in *ski2* mutants (Figures 2D and 3D). The occurrence of transitive siRNAs immediately adjacent to miRNA cleavage sites was similar regardless of the direction of spreading (Figure 5C). Interestingly, of the 21 transcripts targeted by a single miRNA or siRNA, we noticed that in 17 cases, transitive siRNAs mapped to the cleavage fragment least stably base-paired to the miRNA (Figure 5D; Supplementary Table S2). This is significant, because the probability of having at least 17/21 cases right by chance is 0.36% (binomial distribution with basic probability *p* = 0.5 and number of trials *n* = 21). The free energy calculations systematically underestimate the strength of base pairing between 3′-cleavage fragment and 5′-half of the miRNA, because they do not take into account that nucleotides 2–5 of AGO-bound miRNA assume an ordered, helical conformation, thus reducing entropy loss of the miRNA strand upon base pairing (46,47). For this reason, we have counted as correctly predicted those cases where siRNAs accumulate 5′ to the cleavage site, but ΔG for base pairing of a free miRNA to 5′ and 3′ cleavage fragments is similar. Finally, we note that the miRNA targets CSD2 (miR398) and MYB33 (miR159) for which we detected stable 5′-cleavage fragments in *ski2* mutants were not among the 173 transcripts with significantly elevated amounts of siRNAs in *ski2–4/RDR6* compared to SKI2/RDR6 (Supplementary Table S2). We conclude that on many miRNA targets, RDR action results in production of low-abundant siRNAs either 5′ or 3′ to the cleavage site in *ski2–4*. We also conclude that the production of siRNAs correlates poorly with detection of stable 5′-cleavage fragments, and that the strength of cleavage fragment base pairing is a good predictor of the direction of siRNA spreading.

## DISCUSSION

### Degradation of RISC 5′-cleavage fragments is mediated by the SKI2–3–8 complex

5′-cleavage fragments of RISC have been known to turn over rapidly *in planta* since the first observations of miRNA-guided mRNA cleavage (1), but the mechanisms involved have remained ill-defined. This study shows that the *SKI2*, *SKI3* and *SKI8* genes are required for degradation of several RISC 5′-cleavage fragments. Since *SKI2*, *SKI3* and *SKI8* form a cytoplasmic cofactor for the exosome, these observations suggest that, as in *Drosophila* (26), exosomal degradation mediated by the SKI2–3–8 complex is a major degradation pathway of RISC 5′-cleavage fragments in *Arabidopsis*. Nonetheless, the identity of the nucleases involved remains unknown.

### RNA decay pathways and suppression of transitivity

A number of recent studies have demonstrated that plant RNA decay pathways limit RDR6-dependent siRNA production. Mutants in components of the cytoplasmic deadenylation-dependent mRNA decay pathway, nuclear 5′-3′ exonucleolysis, exosome-dependent 3′-5′ exonucleolysis, and non-sense mediated mRNA decay pathway all show enhanced RDR6-dependent siRNA production in transgenic silencing systems and in some cases also from endogenous transcripts (20,23,34,48–51). In a few cases, clear RDR6-dependent phenotypes have also been described, including wax biosynthesis defects in mutants of the core exosome component RRP45B and post-embryonic lethality in *vcs* mutants defective in mRNA decapping, demonstrating that illegitimate transitivity can be detrimental to plant fitness (50,51).

### Implication of SKI2 in suppression of transitivity on miRNA targets

While this manuscript was in preparation, it was shown that mutants in *SKI3* restore RDR6-dependent transgene silencing in hypomorphic *ago1* mutants, and that *ski2*, *ski3* and *xrn4* mutants trigger RDR6-dependent silencing of a 35S*-EIN3* transgene, analogous to enhanced transgene silencing observed in *xrn4* mutants earlier (31,35). The latter study also showed that *xrn4/ski2* double mutants exhibit seedling lethality dependent on RDR6 and DCL2, and that several transcripts, including some miRNA targets, become sources of RDR6-dependent siRNAs in this double mutant background (31). It is of note that miRNA targets constitute only a small fraction of the transcripts that produce illegitimate transitive siRNAs in *xrn4/ski2* (31). In contrast, miRNA targets are strongly enriched in the set of transcripts that produce illegitimate siRNAs in *ski2* single mutants described here. On the other hand, transitive siRNAs are produced in higher abundance in *xrn4/ski2* double mutants than in *ski2* single mutants. These observations suggest that SKI2 may be the primary factor to prevent transitivity on miRNA targets, but that both SKI2- and XRN4-mediated RNA degradation may prevent amplification of transitive siRNAs. The key question therefore becomes what precisely the trigger of transitivity is, and how SKI2 prevents transitivity from being triggered in wild type plants.

### Evidence for aberrant RNAs as triggers of transitivity

The ‘aberrant RNA model’ is typically used to explain secondary siRNA formation, although none of the studies discussed above clearly defines the molecular nature of the trigger of RDR6-dependent siRNA formation. The aberrant RNA model states that defective RNA in sufficient concentrations triggers recognition by RDR6, leading to dsRNA formation and production of siRNAs via Dicer-like ribonucleases. A plausible candidate for an aberrant RNA trigger has recently been identified in transgene silencing. In that system, silencing correlated with presence of an uncapped antisense RNA that may be generated upon cleavage and polyadenylation of un-terminated transcripts from a convergent transcription unit in the T-DNA (52). In general, elevated concentrations of aberrant RNA could be a consequence of either increased frequency of processing errors, or of defective clearance of normal levels of aberrant RNAs, including those produced by RISC cleavage. The aberrant RNA model is therefore consistent not only with recent findings of enhanced transitivity in RNA decay mutants, but also with the fact that mutants in 3′-end formation and splicing enhance RDR6-dependent silencing (53). In particular, this model was proposed to explain the appearance of transitive siRNAs on miRNA targets in *xrn4/ski2* double mutants (31). At first sight, our results on miRNA-triggered transitivity are consistent with the aberrant RNA model, because they clearly show that *SKI2*, *SKI3* and *SKI8* are implicated in degradation of non-polyadenylated RISC 5′-cleavage fragments, and that *ski2–4* produces illegitimate transitive siRNAs. Thus, aberrant RNA in the form of non-polyadenylated 5′-cleavage fragments generated by RISC may trigger transitivity. In particular, this model could explain the production of abundant transitive siRNAs in *ski2–4* covering most of the 5′-, but not the 3′-cleavage fragment of GFP171.

### Evidence against aberrant RNAs as primary triggers of miRNA-induced transitivity

Three observations made in this study are more difficult to reconcile with the aberrant RNA model. First, transitive siRNAs on some targets, including AGO1 and AT5G43740, mapped to the 3′-side of the miRNA cleavage site. Nonetheless, our northern analyses of several miRNA targets, among them AGO1, revealed stabilization exclusively of 5′-cleavage fragments in *ski2* mutants. Second, we noticed that the strength of base pairing between cleavage fragment and miRNA is a good predictor of where transitive siRNAs are produced. It is difficult to explain this observation by a model in which isolated RNA cleavage fragments trigger RDR action and siRNA production. Third, several 5′-cleavage fragments such as CSD2 and MYB33 were easily detectable in *ski2* mutants, yet transitive siRNAs were not detected. Thus, it is appropriate to consider alternatives to the aberrant RNA model for how transitivity can be triggered by miRNAs.

### A ‘RISC trigger’ model for transitivity

To accommodate the observations listed above, an alternative model must assume that the production of transitive siRNAs is triggered at a time where 5′ and 3′-cleavage fragments are still together and base paired to the miRNA. Thus, RISC is likely to be present at the on-set of transitivity. This has previously been proposed in models that suggest the use of RISC-bound small RNA as a primer for RDR6 (54). Such models can only explain the use of the 5′-cleavage fragment as an RDR6 template, however. We propose that RISC is required to initiate secondary siRNA production, and that the dwell time of RISC on a target RNA may be a determinant of whether transitivity is triggered or not. Such dependence on duration of target association may be achieved if RISC interacts only weakly with an RDR6-containing complex. In this case, rapid dissociation of RISC from target transcripts would make RDR6-target mRNA encounters unlikely, analogous to recruitment of RDR only by stalled spliceosomes in *Cryptococcus* (55). In this ‘RISC trigger’ model, SKI2 would catalyze dissociation of RISC from cleaved target RNAs, perhaps by directly using its helicase activity to unwind miRNA-mRNA pairing. Thus, loss of SKI2 helicase activity in the *ski2–4* D472N mutant would allow RDR6 recruitment to cleaved target strands paired to RISC. We note that this model also provides an explanation for the pattern of 5′- and 3′-spreading of siRNAs that we observe. Recent single-molecule fluorescence studies of target cleavage by *Drosophila* Ago2 showed that the target strand least stably base paired to Ago2-bound small RNA leaves first from the RISC-target RNA complex (56). Since transitive siRNAs tend to map to the least stably base paired cleavage fragment in our observations, this suggests that RDR6 normally uses as template the target RNA strand first released after RISC cleavage. Clearly, other factors including presence of RNA secondary structure and RNA binding proteins could also influence template choice of RDR6. We view the RISC trigger model as a useful alternative to the aberrant RNA model in the interpretation of future studies on transitivity, and wish to stress that it should not be considered proven based on the present study alone.

### RDR-dependent siRNA production without RISC?

Although the RISC trigger model states that RISC loaded with a primary small RNA has a more direct role in triggering transitivity than simple generation of aberrant RNA via target cleavage, it does not exclude the possibility that primary siRNAs themselves may derive from RDR action on an aberrant RNA. It is of course formally possible to extend the RISC trigger model to include all types of RDR-dependent siRNA generation if production of minute quantities of RDR-independent siRNAs, akin to centromeric primal siRNAs in *Schizosacharomyces pombe* (57), is postulated to occur. However, there is currently no evidence for such a scenario, and deep sequencing studies of a classical transgene silencing system in *Arabidopsis* failed to reveal read counts of transgene siRNAs in *rdr6* mutants above background levels (52).

### Combining RISC trigger and aberrant RNA models: two waves of transitivity

In the experiments reported by Zhang et al., RDR6-dependent siRNAs were only described in *xrn4/ski2* double mutants, and no particular attention was paid to the distinct patterns of siRNA accumulation observed on different miRNA targets, because both 5′-3′ and 3′-5′ decay pathways were mutated simultaneously (31). In most cases, the siRNA accumulation proximal to the cleavage site that we observe in *ski2–4* was both strongly amplified and extended in *ski2/xrn4* double mutants. This pattern of highly abundant transitive siRNAs covering an extensive region of the miRNA target transcript is similar to what we observe with the GFP171.1 transgene in *ski2–4*. It is possible that defective RNA degradation amplifies transitivity, such that production of abundant transitive siRNAs involves multiple rounds of amplification: The actual trigger event produces low-abundant secondary siRNAs in close proximity to the cleavage site. In turn, presence of sufficient amounts of aberrant target RNA (e.g. cleavage fragments of endogenous miRNA targets in *xrn4/ski2* or of 35S::GFP171.1 in *ski2–4*) promotes reiterated amplification of these siRNAs to produce highly abundant siRNAs, possibly still dependent on RISC. This distinction between a trigger wave and amplification waves is consistent with two genetic observations that remain unexplained. First, the helicase SDE3 is required for transgene silencing in systems strongly dependent on siRNA amplification via RDR6 (18,58,59). It is not, however, required for production of RDR6-dependent *trans-*acting siRNAs (tasiRNAs), a class of phased siRNAs that must result from a single amplification wave of a miRNA-guided cleavage fragment, because reiterated amplification would disrupt their phased pattern of accumulation (59–61). Second, mutant alleles of RDR6 itself exist that allow tasiRNA production, but not transgene silencing (62). They may therefore genetically uncouple trigger and amplification waves of transitivity. We note that the two-step RISC trigger model is very similar to a model recently proposed to explain piRNA-mediated production of transgenerationally maintained siRNAs in *C.elegans*. In this system, the primary trigger (piRNA) leads to production of AGO-dependent, low-abundant secondary siRNAs that map close to the piRNA cleavage site. These siRNAs are amplified via the action of the nuclear RNAi pathway that includes dedicated AGO proteins to produce abundant siRNAs along the entire target transcript (63). Thus, a two-step mechanism may be fundamental to how small silencing RNAs are amplified by AGOs and RDRs.

## ACCESSION NUMBERS

Small RNA sequence data summarized in Figures 4B and 5 and Supplementary Tables S2 and S3 have been submitted to the European Nucleotide Archive (ENA) under accession number E-MTAB-3781.

## Supplementary Material

SUPPLEMENTARY DATA
