# Supplementary material for: SKI2 mediates degradation of RISC 5′-cleavage fragments and prevents secondary siRNA production from miRNA targets in Arabidopsis
Source: Nucleic Acids Res. 2015 Oct 12;43(22):10975–88. doi: 10.1093/nar/gkv1014 (PMC4678812; doi:10.1093/nar/gkv1014)
Supplement: SUPPLEMENTARY DATA [file supp_43_22_10975__index.html]

SKI2 mediates degradation of RISC 5′-cleavage fragments and prevents secondary siRNA production from miRNA targets in Arabidopsis — SUPPLEMENTARY DATA 

# SKI2 mediates degradation of RISC 5′-cleavage fragments and prevents secondary siRNA production from miRNA targets in *Arabidopsis*

## SUPPLEMENTARY DATA

- SUPPLEMENTARY DATA
