# Supplementary material for: SKI2 mediates degradation of RISC 5′-cleavage fragments and prevents secondary siRNA production from miRNA targets in Arabidopsis
Source: Nucleic Acids Res. 2015 Oct 12;43(22):10975–88. doi: 10.1093/nar/gkv1014 (PMC4678812; doi:10.1093/nar/gkv1014)
Supplement: SUPPLEMENTARY DATA [file supp_gkv1014_nar-01716-y-2015-File008.pdf]

Figure S1

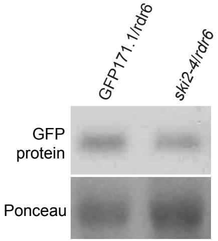

Figure S2: SNP detection in *ski2-4.*

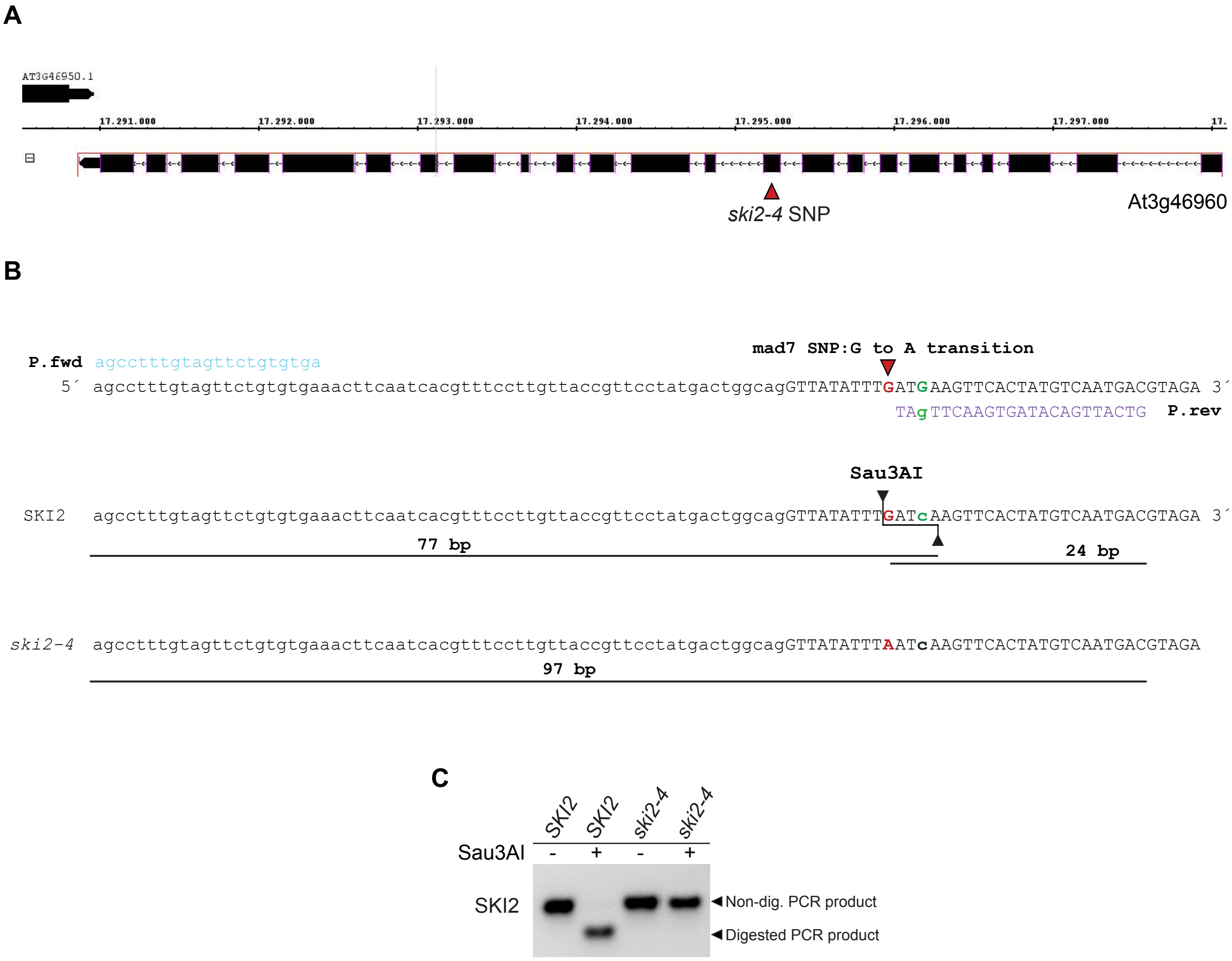

Figure S3. Genotyping of *ski3-5 and ski8-1* T-DNA insertion mutants.

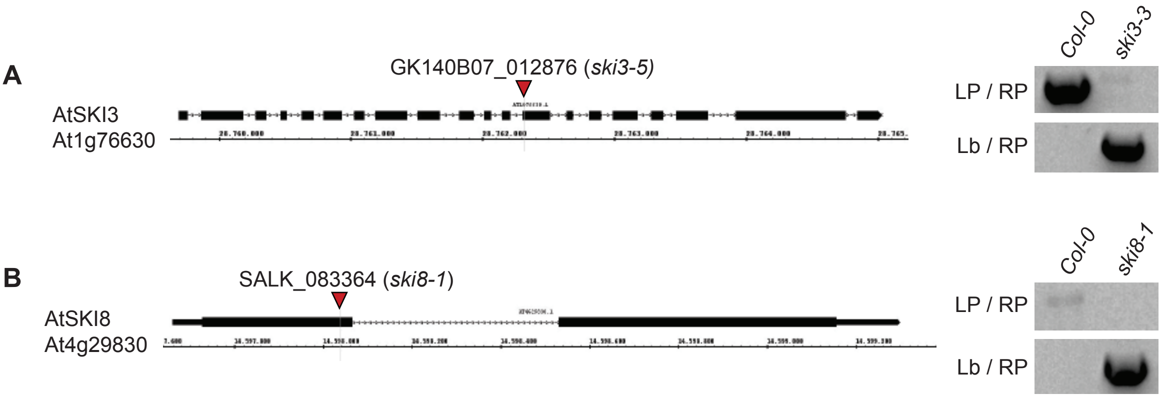

Suppl. Table 1. Filtered list of candidate genes exhibiting SNPs within a peak distance of 100000 bp

Chromosome	Genome position_TAIR8	Gene accession	Ref_Base	Called_Base	Peak_distance	Coverage	Concordance	Number of aa*	Ref_ aa*	Called_ aa*
3	17246790	AT3G46800	C	T	5947	6	1	738	A	A
3	17229243	AT3G46740	C	T	11600	93	1	1039	V	M
3	17254124	AT3G46820	C	T	13281	78	0.99	332	C	Y
3	17224586	AT3G46730	C	T	16257	20	0.95	2012	S	N
3	17222504	AT3G46720	C	T	18339	37	1	755	S	N
3	17222488	AT3G46720	C	T	18355	43	1	771	L	L
3	17222378	AT3G46720	G	C	18465	50	1	881	P	R
3	17262698	AT3G46840	C	T	21855	4	1	363	G	G
3	17262728	AT3G46840	T	C	21885	9	1	393	N	N
3	17262729	AT3G46840	A	G	21886	10	1	394	T	A
3	17206656	AT3G46680	C	G	34187	80	1	997	A	P
3	17277550	AT3G46890	C	T	36707	60	0.97	466	A	T
3	17197949	AT3G46650	G	A	42894	35	1	714	C	C
3	17290184	AT3G46910	G	T	49341	10	1	400	V	L
3	17306264	AT3G46960	C	T	65421	95	1	1387	D	N
3	17323547	AT3G46990	C	T	82704	80	1	662	S	L
3	17332049	AT3G47020	A	G	91206	3	1	929	E	G
3	17142644	AT3G46530	A	T	98199	7	0.86	1588	F	I
3	17350990	AT3G47080	G	C	110147	3	1	125	A	G

* aa: amino acid

Table S2. Known miRNA/siRNA targets with higher siRNA abundance in *ski2-4/RDR6* than in *SKI2/RDR6*

Locus Identifier	Gene annotation	miRNA/siRNA	RDR6-dep.	Cleavage site	5'siRNAs	3'siRNAs	ΔG 5'CF	ΔG 3'CF
AT1G62670	RNA PROCESSING FACTOR 2 (RPF2)	miR161.1, miR161.2, miR400	YES		*	*		
AT1G62930	RNA PROCESSING FACTOR 3 (RPF3)	miR161.1, miR161.2, miR400	YES		*	*		
AT1G62910	PPR superfamily protein	miR161.1, miR161.2, miR400, TAS2	YES		*	*		
AT1G63150	PPR superfamily protein	miR161.1, miR161.2, miR400, TAS2	YES		*	*		
AT1G63080	PPR superfamily protein	miR161.1, miR161.2, miR400, TAS2	YES		*	*		
AT1G63130	PPR superfamily protein	miR161.1, miR161.2, miR400, TAS2	YES		*	*		
AT1G63400	PPR superfamily protein	miR161.1, miR161.2, miR400, TAS2	YES		*	*		
AT1G63330	PPR superfamily protein	miR161.2, miR400, TAS1a, TAS2	YES		*	*		
AT1G62590	PPR superfamily protein	miR161.2, miR400, TAS1c, TAS2	YES		*	*		
AT1G63070	PPR superfamily protein	miR161.2, miR400, TAS2	YES		*	*		
AT1G12775	PPR superfamily protein	TAS1a, TAS2	YES		*	*		
AT1G12620	PPR superfamily protein	TAS2	YES	4295284	YES	NO	-17.6	-9.1
AT5G43270	Squamosa promoter binding protein-like 2 (SPL2)	miR156	YES	17360766	YES	NO	-13.8	-16.9
AT2G28350	AUXIN RESPONSE FACTOR10 (ARF10)	miR160	YES	12115750	YES	NO	-13.8	-22.2
AT1G77850	AUXIN RESPONSE FACTOR17 (ARF17)	miR160	YES	29274198	YES	NO	-20.2	-22.2
AT5G39610	NAC domain containing protein 6 (NAC6)	miR164	YES	15858805	NO	YES	-12.5	-18.7
AT2G34710	PHABULOSA (PHB)	miR165	YES	14642656	YES	NO	-16.2	-19.9
AT1G30490	PHAVOLUTA (PHV)	miR165	YES	10799339	YES	NO	-16.2	-19.9
AT5G60690	REVOLUTA (REV)	miR165	YES	24398887	YES	NO	-16.2	-19.9
AT1G30330	AUXIN RESPONSE FACTOR6 (ARF6)	miR167	YES	10686487	YES	NO	-13.2	-18.1
AT1G48410	ARGONAUTE1 (AGO1)	miR168	YES	17891549	NO	YES	-15.6	-12.3
AT2G45160	LOST MERISTEMS 1 (LOM1)	miR171a<br>miR171b<br>miR171c<br>miR170	YES<br>YES<br>YES<br>YES	18619139<br>18619139<br>18619139<br>18619139	YES<br>YES<br>YES<br>YES	NO<br>NO<br>NO<br>NO	-19.8<br>-11.9<br>-11.9<br>-14.0	-18.0<br>-18.2<br>-18.2<br>-18.0
AT4G36920	APETALA 2 (AP2)	miR172	YES	17403028	YES	NO	-19.0	-14.4
AT3G15030	TCP family transcription factor 4 (TCP4)	miR319	YES	5063309	YES	NO	-16.2	-16.7
AT4G18390	TCP family transcription factor 2 (TCP2)	miR319	YES	10163410	YES	NO	-12.3	-16.7
AT1G53230	TCP family transcription factor 3 (TCP3)	miR319	YES	19850475	YES	NO	-16.2	-16.4
AT3G23690	bHLH DNA-binding superfamily protein	miR393	YES	8529821	YES	(YES)	-16.4	-16.5

Locus Identifier	Gene annotation	miRNA/siRNA	RDR6-dep.	Cleavage site	5'siRNAs	3'siRNAs	ΔG 5’CF	ΔG 3’CF
AT1G12820	AUXIN SIGNALING F-BOX 3 (AFB3)	miR393	YES	4369093	NO	YES	-14.1	-18.6
AT2G33770	PHOSPHATE 2 (PHO2)	miR399	NO	14281779	YES	NO	-18.5	-15.3
			NO	14281912	YES	NO	-15.3	-18.6
			NO	14282001	YES	NO	-17.0	-18.6
			NO	14282058	YES	NO	-10.0	-18.6
			NO	14282115	YES	NO	-11.0	-18.6
AT1G12290	Disease resistance protein	miR472	YES	4180693	NO	YES	-18.9	-13.2
AT5G43740	Disease resistance protein	miR472	YES	17566551	NO	YES	-19.3	-13.2
AT1G02860	Nitrogen limitation adaptation (NLA)	miR827	NO	635410	YES	NO	-13.2	-15.8
AT3G21170	F-box protein	miR859	NO	7420705	*	*	-11.1	-16.7

Table S3. Other targets with higher siRNA abundance in ski2-4 vs. WT

Locus Identifier	Gene model description
AT1G06148	Unknown
AT3G44630	Disease resistance protein (TIR-NBS-LRR class) family
AT4G03380	Unknown
AT1G62914	PPR protein
AT3G09440	Heat shock protein 70 (Hsp 70) family protein
AT4G20320	CTP synthase family protein
AT5G35610	PAH2 superfamily protein
AT1G35710	LRR protein kinas e family protein
AT2G18530	Protein kinase superfamily protein
AT4G27930	Unknown
AT2G05635	DEAD helicase RAD3/XP-D subfamily protein
AT1G49260	Unknown
AT4G08990	DNA-Methyltransferase family protein
AT3G61700	Unknown
AT5G45950	GDSL-like lipase
AT5G38370	Zinc-finger protein
AT3G28520	ATP hydrolase family protein
AT1G11370	Pectin lyase-like superfamily protein
AT1G17780	Unknown
AT3G26870	S1 family protein
AT2G24340	bZIP transcription factor family protein
AT2G16580	SAUR-like auxin-responsive protein family
AT1G60400	F-box/RNI-like superfamily protein
AT3G66654	Isomerase family protein
AT1G24570	Unknown
AT2G03370	Glycosyltransferase family protein
AT5G02170	Transmembrane amino acid transporter family protein
AT5G54940	Translation initiation factor SUI1 family protein
AT5G48860	Unknown
AT2G16620	Protein kinase superfamily protein
AT1G48912	Unknown
AT4G24974	S1 family protein
AT4G08530	PR protein
AT3G50320	Unknown
AT5G60390	Elongation factor Tu family protein
AT3G15960	Mismatched DNA-binding protein
AT3G16930	Unknown
AT4G03580	Cysteine proteinases superfamily protein
AT5G53610	Unknown
AT4G16040	Unknown
AT5G60080	Protein kinase superfamily protein
AT4G13885	RNase H-like superfamily protein
AT1G33070	MADS-box family protein
AT2G02440	Unknown
AT2G15260	RING/U-box superfamily protein
AT5G56070	Unknown
AT1G61230	Mannose-binding lectin superfamily protein
AT5G22860	Serine-type peptidase protein
AT4G17410	Zinc finger protein

Locus Identifier	Gene model description
AT4G26460	SAM-dependent methyltransferase superfamily protein
AT1G22930	Unknown
AT4G08395	Unknown
AT3G60750	Transketolase family protein
AT1G22120	Unknown
AT2G02835	Unknown
AT3G50400	GDSL-like lipase family protein
AT2G01780	Curculin-like (mannose-binding) lectin family protein
AT3G61028	Unknown
AT1G20405	Unknown
AT2G16365	F-box family protein
AT4G36590	MADS-box family protein
AT1G13760	Unknown
AT2G20597	Thionin family protein
AT1G02470	Lipid transport superfamily protein
AT1G54410	Dehydrin family protein
AT1G80220	Zinc finger protein
AT1G53640	Unknown
AT1G10586	bHLH- DNA-binding superfamily protein
AT1G77100	Peroxidase superfamily protein
AT2G21235	bZIP transcription factor family protein
AT4G24600	Unknown
AT3G48770	DNA / ATP binding protein
AT2G16575	Unknown
AT4G02480	ATP hydrolase family protein
AT5G02490	(Hsp70-2)
AT5G27360	(SFP2)
AT5G50480	Nuclear factor y, subunit C6 (NF-YC6)
AT5G60440	Agamous-like 62 (AGL62)
AT3G66656	Agamous-like 91 (AGL91)
AT2G14890	Arabinogalactan protein 9 (AGP9)
AT3G02020	Aspartate kinase 3 (AK3)
AT4G29900	Autoinhibited Ca(2^+)-ATPASE 10 (ACA10)
AT1G35520	Auxin response factor 15 (ARF15)
AT2G22850	Basic leucine-zipper 6 (bZIP6)
AT3G62420	Basic region/leucine zipper motif 53 (BZIP53)
AT1G52400	Beta glucosidase 18 (BGLU18)
AT1G55120	Beta-fructofuranosidase 5 (FRUCT5)
AT4G21670	C-terminal domain phosphatase-like 1 (CPL1)
AT1G20620	Catalase 3 (CAT3)
AT4G33270	Cell division cycle 20.1 (CDC20.1)
AT1G29930	Chlorophyll a/b binding protein 1 (CAB1)
AT3G62422	Conserved peptide upstream open reading frame 3 (CPuORF3)
AT3G02468	Conserved peptide upstream open reading frame 9 (CPuORF9)
AT5G53560	Cytochrome b5 isoform E (CB5-E)
AT5G57800	Eceriferum 3 (CER3)
AT1G54040	Epithiospecifier protein (ESP)
AT1G28370	ERF domain protein 11 (ERF11)
AT1G47128	Esponsive to dehydration 21A (RD21A)
AT3G47340	Glutamine-dependent asparagine synthase 1 (ASN1)
AT2G21660	Glycine-rich rna-binding protein 7 (GRP7)

Locus Identifier	Gene model description
AT4G09740	Glycosyl hydrolase 9B14 (GH9B14)
AT4G23560	Glycosyl hydrolase 9B15 (GH9B15)
AT5G56030	Heat shock protein 81-2 (HSP81-2)
AT5G56010	Heat shock protein 81-3 (HSP81-3)
AT5G56000	Heat shock protein 81.4 (Hsp81.4)
AT2G32370	Homeodomain glabrous 3 (HDG3)
AT3G08940	Light harvesting complex photosystem II (LHCB4.2)
AT2G15050	Lipid transfer protein (LTP)
AT2G38540	Lipid transfer protein 1 (LP1)
AT3G45140	Lipoxygenase 2 (LOX2)
AT4G22700	Lob domain-containing protein 32 (LBD32)
AT1G56070	Low expression of osmotically responsive genes 1 (LOS1)
AT3G23167	Low-molecular-weight cysteine-rich 39 (LCR39)
AT2G14935	Low-molecular-weight cysteine-rich 40 (LCR40)
AT4G04840	Methionine sulfoxide reductase B6 (MSRB6)
AT2G19800	Myo-inositol oxygenase 2 (MIOX2)
AT4G37590	Naked pins in yuc mutants 5 (NPY5)
AT4G24020	Nin-like protein 7 (NLP7)
AT4G13250	Non-yellow coloring 1 (NYC1)
AT2G34420	Photosystem ii light harvesting complex gene B1B2 (LHB1B2)
AT1G14870	Plant cadmium resistance 2 (PCR2)
AT4G05320	Polyubiquitin 10 (UBQ10)
AT2G42840	Protodermal factor 1 (PDF1)
AT3G44480	Recognition of peronospora parasitica 1 (RPP1)
AT2G27070	Response regulator 13 (RR13)
AT5G07210	Response regulator 21 (RR21)
AT3G23810	S-Adenosyl-L-Homocysteine (SAH) hydrolase 2 (SAHH2)
AT3G02470	S-Adenosylmethionine decarboxylase (SAMDC)
AT3G10985	Senescence associated gene 20 (SAG20)
AT5G22980	Serine carboxypeptidase-like 47 (scpl47)
AT5G48600	Structural maintenance of chromosome 3 (SMC3)
AT4G34980	Subtilisin-like serine protease 2 (SLP2)
AT1G66570	Sucrose-proton symporter 7 (SUC7)
AT3G26520	Tonoplast intrinsic protein 2 (TIP2)
AT4G27550	Trehalose-6-phosphatase synthase S4 (TPS4)
AT1G16980	Trehalose-phosphatase/synthase 2 (TPS2)
AT4G03560	Two-pore channel 1 (TPC1)
AT1G55860	Ubiquitin protein ligase 1 (UPL1)
AT1G48910	Yucca 10 (YUC10)
AT2G32930	Zinc finger nuclease 2 (ZFN2)

Table S3. Continued

Table S4. Oligonucleotides

Oligoname	Sequence (5´to 3´)	Purpose
mad7_genot_Sau3AI_F	CGTCATTGACATAGTGAACTTGAT	Genotyping PCR
mad7_genot_Sau3AI_R	AGCCTTTGTAGTTCTGTGTGA	Genotyping PCR
RDR6.sde1_F	GAGGTGGCCTCCCCGTATCC	Genotyping PCR
RDR6.sde1_R	GGCAATAGGCTCTGGTTCGGG	Genotyping PCR
Lbb1.3_Salk	ATTTTGCCGATTTCGGAAC	Genotyping PCR
ski8-1(VIP3)_Salk LP	ACAGAGAGACCACGAGAGCAG	Genotyping PCR
ski8-1(VIP3)_Salk RP	GAAGCAAATAAAAACTCCACTGC	Genotyping PCR
ski2-5_Salk_LP	GAACTGGTCTTTTTGTCGTGC	Genotyping PCR
ski2-5_Salk RP	TAAATTTGCGGACATTTGAGG	Genotyping PCR
ski3-2_Salk LP	AGATGAGGCTTTTGAGAGTT	Genotyping PCR
ski3-2_Salk RP	ATTAGCGCATTCCATAACAGATTC	Genotyping PCR
GK_T-DNA_F	ATAATAACGCTGCGGACATCTACATTTT	Genotyping PCR
mGFP7_genot_F	GATGGCCCTGTCCTTTTAC	Genotyping PCR
XD216_genot_R	GGGGATCCTGATTGAGCCG	Genotyping PCR
ski2-2.LP	AGGTATCTCAATGTCCGGACC	Genotyping PCR
ski2-2.RP	ACACAACCGTGGCACTTATTC	Genotyping PCR
AGO1_5´_F	AGAGAAGAACGATGCTCCA	probe
AGO1_5´_R	TTGTTGCTGTTGTGGTGGTT	probe
AGO1_3´_F	GGATTTGCACCATTATGAT	probe
AGO1_3´_R	TCAAGAACCTGCAGAGCTT	probe
GFP1_5´probe_F	AGTAAAGGAGAAGAACTTTTCACT	probe
GFP4_5´probe_R	TTCCGTCCTCCTTGAAATCGA	probe
35S-Ter_probe_F	GATATTGGCGCGGCTCAATCA	probe
35S-Ter_probe_R	AGGGTTTCTTATATGCTCAACACA	probe
LOM2 5´probe_F	AATGCGACCAAATGGGTTTC	probe
LOM2 5´probe_R	TGTTATCTCCGCCGTTTGTG	probe
LOM2 3´probe_F	TTCGGATCGCAGCTTACAGA	probe
LOM2 3´probe_R	GCGCGTTAATCACACCGTTA	probe
CSD2_5´probe2.F	CCAAACGTCAAACATAGCAGCA	probe
CSD2_5´probe2.R	CCGCGGAAACAACTGTCAAC	probe
CSD2_3´probe2.F	GGGTGACCTGGGAAACATAA	probe
CSD2_3´probe2.R	TCAAGCCAATCACACCACAT	probe
MYB33_5´_F	AAGCGACTTTGGGAATCTGA	probe
MYB33_5´_R	AGGAACAATGCCATCCGTAG	probe
MYB33_3´_F	CACCAAGGCAGAGAGAAAAAAAGCG	probe
MYB33_3´_R	ACAGGTGGCATGTTGCTCCAAGAAC	probe
3´RNA-adapter	P-CUAGAUGAGACCGUCGACAUGAAUUC-NH2	3´RACE
anchor rv1	GAATTCATGTCGACGGTCTCA	3´RACE
anchor rv2	CATGTCGACGGTCTCATCTAG	3´RACE
GFP13_fwd	TTTGCACTACTGGAAAACTACC	3´RACE
GFP11_fwd	ATGGCCCTGTCCTTTTACC	3´RACE

SUPPLEMENTARY FIGURE LEGENDS

Figure S1. GFP protein levels in *ski2-4/rdr6* seedlings

Western blot analysis of GFP protein extracted from seedlings of GFP171.1 and *ski2-4/rdr6*

Figure S2. Validation of *ski2-4* single nucleotide polymorphism.

A. SKI2 gene model. A red arrow indicates the position of the *ski2-4* SNP.
B. Partial genomic sequence of SKI2 and PCR strategy for *ski2-4* SNP validation. The reverse primer flanks the SNP and was designed to introduce a point mutation in order to generate a Sau3AI restriction site (GATC) in the wild type allele, but not in the mutant allele.
C. Gel analysis of GFP171.1 and *ski2-4* PCR product and Sau3AI restriction digest. For the wild type allele, two fragments in the size of 77 bp and 24 bp are expected, whereas the mutant allele remains uncleaved (97 bp).

Figure S3. Gene models and genotyping of *ski3-5*, *ski8-1* and *hen1-6* T-DNA insertional mutants.

A. SKI3 gene model and PCR validation of *ski3-5* T-DNA insertional mutant. The genomic position of T-DNA insertion is indicated by a red arrow.
B. SKI8 gene model and PCR validation of *ski8-1* T-DNA insertional mutant. The genomic position of T-DNA insertion is indicated by a red arrow.

Table S1: Filtered list of candidate genes exhibiting SNPs within a distance of 100000 bp from the peak.

Table S2: Genes known to be miRNA or siRNA targets with significantly higher siRNA levels in *ski2-4/RDR6* than in *SKI2/RDR6*. For each gene, the coordinates (TAIR9/10) of the cleavage site are given, and it is noted whether siRNAs are RDR6-dependent, and whether they match the 5'-cleavage fragment (5'-CF, 5'siRNAs) or the 3'-cleavage fragment (3'-CF, 3'siRNAs). Free energies of base pairing between miRNA and cleavage fragment are also listed. Asterisks denote cases where entire transcripts are covered in abundant siRNAs. Since these cases are qualitatively different from the typical peaks proximal to the cleavage site, they are excluded from the analysis. Examples that follow the base pairing rule are in black, examples that violate it are in red. miR399/PHO2 is counted among the correctly predicted cases, because 4 out of 5 miR399 sites show a pattern of strength of base pairing consistent with the rule. For miR393/AT3G23690, (YES) in the 3'siRNA column indicates that

a few siRNA reads are detected 3’ to the cleavage site. Since the main siRNA peak is 5’ to the cleavage site, this case is also counted among the correctly predicted ones.

Table S3: Loci not annotated as miRNA targets with significantly higher siRNA levels in *ski2-4/RDR6* than in *SKI2/RDR6*.

Table S4: Oligonucleotides used in this study.
